# Canadian polar bear population structure using genome‐wide markers

**DOI:** 10.1002/ece3.6159

**Published:** 2020-03-24

**Authors:** Evelyn L. Jensen, Christina Tschritter, Peter V. C. de Groot, Kristen M. Hayward, Marsha Branigan, Markus Dyck, Rute B. G. Clemente‐Carvalho, Stephen C. Lougheed

**Affiliations:** ^1^ Department of Biology Queen’s University Kingston ON Canada; ^2^ Department of Environment and Natural Resources Government of the Northwest Territories Inuvik NT Canada; ^3^ Department of Environment Government of Nunavut Igloolik NU Canada; ^4^ Present address: Department of Ecology and Evolutionary Biology Yale University New Haven CT USA

**Keywords:** Arctic, conservation, ddRAD, population genetics, single nucleotide polymorphism, *Ursus maritimus*

## Abstract

Predicting the consequences of environmental changes, including human‐mediated climate change on species, requires that we quantify range‐wide patterns of genetic diversity and identify the ecological, environmental, and historical factors that have contributed to it. Here, we generate baseline data on polar bear population structure across most Canadian subpopulations (*n* = 358) using 13,488 genome‐wide single nucleotide polymorphisms (SNPs) identified with double‐digest restriction site‐associated DNA sequencing (ddRAD). Our ddRAD dataset showed three genetic clusters in the sampled Canadian range, congruent with previous studies based on microsatellites across the same regions; however, due to a lack of sampling in Norwegian Bay, we were unable to confirm the existence of a unique cluster in that subpopulation. These data on the genetic structure of polar bears using SNPs provide a detailed baseline against which future shifts in population structure can be assessed, and opportunities to develop new noninvasive tools for monitoring polar bears across their range.

## Graphical abstract

With ongoing rapid sea ice loss and environmental change in the Arctic, there is the potential for rapid changes in polar bear population structure. Here, we generated baseline data on polar bear population structure across 12 of 13 Canadian subpopulations using genome‐wide SNPs. These data on the genetic structure of polar bears using SNPs provide a detailed baseline against which future shifts in population structure can be assessed, and opportunities to develop new noninvasive tools for monitoring polar bears across their range.

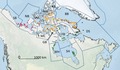

## 1 INTRODUCTION

Contemporary genetic population structure reflects the interplay between genetic drift, selection, and gene flow, which is modulated by climate, landscape, and population history. Predicting how species might respond to future environments requires that we identify the factors that have shaped present‐day intraspecific genetic structure (Davis & Shaw, 2001; Schierenbeck, 2017). The consequences of human‐mediated environmental change (e.g., climate change, habitat degradation, and fragmentation) include poleward or altitudinal range shifts (Chen, Hill, Ohlemuller, Roy, & Thomas, 2011; Parmesan et al., 1999), isolation among previously contiguous populations (e.g., Row et al., 2011), and loss of genetic diversity (e.g., Rubidge et al., 2012). The sensitive ecosystems and biodiversity of the Arctic are particularly susceptible to climate change as northern regions are warming at a rate greater than twice the global average (Comiso & Hall, 2014). Recent climate projections suggest that the Arctic could experience ice‐free summers as soon as 2030 (Overland & Wang, 2013), leading to the question of what consequences an ice‐free Arctic will have on the population structure of ice‐adapted species such as the polar bear.

Our current understanding of polar bear (*Ursus maritimus*) population structure has emerged over time through population genetic studies based on mitochondrial sequences and microsatellite genotypic data (Malenfant, Davis, Cullingham, & Coltman, 2016; Paetkau et al., 1999; Paetkau, Calvert, Stirling, & Strobeck, 1995; Peacock et al., 2015). A recently developed SNP chip for polar bears (Malenfant, Coltman, & Davis, 2015), used for a preliminary population structure analysis, revealed four genetic clusters in the Canadian Arctic; however, other than this no large‐scale study has yet been published using genome‐wide markers, although there are focused studies at more local levels (Malenfant, Davis, Richardson, Lunn, & Coltman, 2018; Viengkone et al., 2016). In general, polar bears show a pattern of isolation‐by‐distance at both population and individual levels (Campagna et al., 2013; Paetkau et al., 1999) within these distinct genetic clusters in the Canadian Arctic (Malenfant et al., 2016; Paetkau et al., 1999).

With ongoing rapid sea ice loss and environmental change in the Arctic (Comiso, 2002, 2012; Howell, Duguay, & Markus, 2009; Howell, Tivy, Yackel, & McCourt, 2008; Rothrock, Yu, & Maykut, 1999), there is the potential for rapid changes in polar bear population structure (Hamilton & Derocher, 2019; Laidre et al., 2015; Vongraven & Peacock, 2012). Responses of polar bears to climate change are not likely to be uniform across their range (Rode et al., 2014). For example, some telemetry studies show increased frequency of long‐distance swimming in response to unsuitable ice coverage for travel and hunting (Durner et al., 2011; Pagano, Durner, Amstrup, Simac, & York, 2012). In contrast, a recent study based on telemetry and genetic data identified range size contractions in Baffin Bay, suggesting increasing physical and genetic isolation of this subpopulation (Laidre et al., 2018).

Here, we aim to further develop our understanding of polar bear population structure in Canada using a new set of SNP markers developed through double‐digest restriction site‐associated DNA (ddRAD) sequencing. We compare our results based on analyses of the ddRAD dataset to previous studies of polar bear population structure.

## 2 METHODS

### 2.1 Sample collection and DNA extraction

**FIGURE 1:**
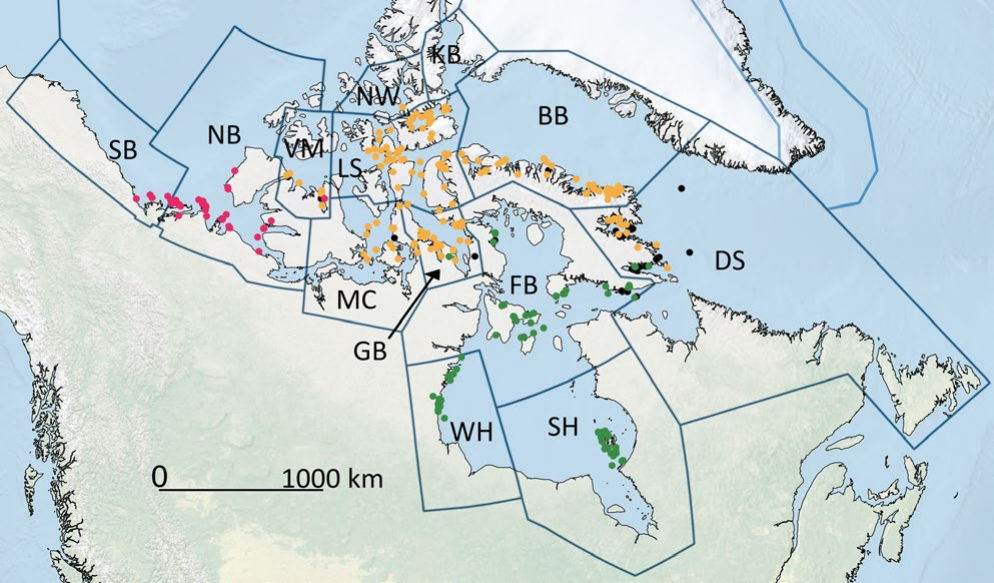
Map of samples used for analyses. Outlined regions are the subpopulations that are wholly or partially in Canada; abbreviations follow Table 1. Colored points correspond to the sampling location and genetic cluster that the individual has majority assignment to, based on the SNP dataset and STRUCTURE analysis (pink = Polar Basin, orange = Arctic Archipelago, green = Hudson Complex). Individuals with membership of <0.7 to a cluster are represented as black dots

The pan‐Arctic geographic range of polar bears is divided into 19 subpopulations or “management units” (Durner, Laidre, & York, 2018), 13 of which are wholly or partially within Canada (Figure 1). Our work draws upon a set of archived muscle tissue samples (dry or frozen in ethanol) accumulated by the Nunavut and Northwest Territories governments from polar bears harvested by Inuit hunters, in line with annual harvest regulations. Most of these tissue samples have not been used in previous studies and represent an independent sample from which to draw inferences. We sought a balanced number of samples from each subpopulation, while ensuring that they were collected in as close to the same period of time as possible. We used samples from 12 of the 13 subpopulations that occur in Canada. For ten subpopulations, we had at least 11 sampled individuals (range: 11–59), with only five samples and one sample from Southern Beaufort Sea and Norwegian Bay, respectively, and no samples from the Kane Basin subpopulation (Table 1). While this sampling limitation is important, we note that these latter two subpopulations are estimated to have very few individuals (KB = 357, NW = 203—see Hamilton & Derocher, 2019). To minimize sampling confounds, we selected samples spanning a minimum breadth of years, so the mean sampling dates ranged from 2008 (Gulf of Boothia) to 2014 (Davis Strait), with the exception of our single Norwegian Bay sample that was collected in 2004.

**TABLE 1:** Diversity metrics of each of the surveyed Canadian polar bear subpopulations based on the single nucleotide polymorphisms dataset (13,488 loci)

Subpopulation	*n*	*H*_O_	*H*_E_	*G*_IS_	Self‐assignment	Main genetic cluster
Baffin Bay (BB)	42	0.19	0.18	−0.04	0.73	Arctic Archipelago
Davis Strait (DS)	36	0.19	0.18	−0.02	0.64	Arctic Archipelago/Hudson Complex
Foxe Basin (FB)	41	0.18	0.17	−0.03	0.63	Hudson Complex
Gulf of Boothia (GB)	36	0.19	0.18	−0.01	0.53	Arctic Archipelago
Lancaster Sound (LS)	59	0.19	0.19	−0.03	0.78	Arctic Archipelago
M’Clintock Channel (MC)	19	0.19	0.18	−0.04	0.46	Arctic Archipelago
Northern Beaufort Sea (NB)	33	0.2	0.19	−0.05	0.93	Polar Basin
Norwegian Bay (NW)	1	—	—	—	—	Arctic Archipelago
Southern Beaufort Sea (SB)	5	0.2	0.18	−0.08	—	Polar Basin
Southern Hudson Bay (SH)	39	0.18	0.17	−0.04	0.68	Hudson Complex
Viscount Melville Sound (VM)	11	0.19	0.18	−0.04	0.04	Arctic Archipelago/Polar Basin
Western Hudson Bay (WH)	36	0.18	0.17	−0.04	0.37	Hudson Complex

Abbreviations: *G*_IS_, inbreeding coefficient; *H*_E_, expected heterozygosity; *H*_O_, observed heterozygosity; *n*, sample size; self‐assignment, the mean assignment rate of individuals back to their subpopulation of sampling.

Genomic DNA was extracted from these samples using a modified salt extraction protocol (Aljanabi & Martinez, 1997), with the addition of RNase A (Thermo Fisher Scientific) following the lysis step. DNA quality was assessed by running it on 1.5% agarose gels stained with RedSafe^TM^ Nucleic Acid Staining Solution (iNtRON Biotechnology). Only extractions with high molecular weight DNA were used for library preparation. Extracts were quantified using a NanoDrop ND_1000 Spectrophotometer (NanoDrop Technologies Inc.).

### 2.2 Double‐digest restriction site‐associated DNA sequencing

We chose to use ddRAD to discover and genotype a new panel of SNP loci, rather than to use the existing SNP chip. The chip has 3,411 putatively neutral SNPs developed based on RAD sequencing of 38 individuals (Malenfant et al., 2015); electing to use ddRAD allowed us to target more loci while possibly reducing ascertainment bias. We constructed ddRAD libraries for 528 individuals following the Peterson, Weber, Kay, Fisher and Hoekstra (2012) protocol, with the addition of adapters that were modified to have degenerate base regions (Schweyen, Rozenberg, & Leese, 2014; Vendrami et al., 2017) to allow for removal of PCR duplicates using bioinformatic tools. We used 1,000 ng of total genomic DNA for each individual, and digested it with MluCI and PstI restriction enzymes (New England Biolabs), before pooling 34–46 uniquely barcoded individuals at a time, and size selecting to 400–490 bp (DNA insert size of 275–365 bp) using a BluePippin Prep (Sage Science) with a 2% agarose cassette. Library pools were amplified using 10–14 cycles in eight parallel PCRs. Libraries were sequenced using one lane each of paired‐end 125 bp Illumina HiSeq 2500 at The Centre for Applied Genomics (SickKids Hospital). Technical replicates were included in each library to assess genotyping error rates.

### 2.3 Sequence assembly, variant detection, and SNP filtering

After assessing the quality of each sequencing run, the data were processed as follows. First, read duplicates were removed using a custom script developed by E. Jensen (https://github.com/Eljensen/ParseDBR_ddRAD). Briefly, the script removes duplicates when the degenerate region in the P2 index matches between reads to ensure PCR duplicates are removed. Once this is done, libraries were demultiplexed using the “process_radtags” tool included as part of Stacks v2.2 (Catchen, Hohenlohe, Bassham, Amores, & Cresko, 2013). Because the barcodes are part of the sequence in both reads, “process_radtags” was run using the “inline_inline” mode. Additionally, the “clean” functionality was activated to remove reads with uncalled bases, and the “rescue” functionality was used to rescue barcodes and RAD‐tags.

Reads from demultiplexed samples were then individually aligned to the Polar Bear reference genome (assembly version UrsMar_1.0) [PMID: 24813606] using the BWA‐MEM v0.7.17 aligner (Li & Durbin, 2009), excluding reads with a minimum quality score of <30. Alignments were sorted, indexed, and read pairs were fixed using tools from the SAMtools v1.9 suite (Li et al., 2009).

Finally, the alignments were used to call SNPs. To reduce the computation time, we used an initial representative group of 327 individuals from the first set of libraries sequenced for variant detection using BCFTOOLS v1.9 (Danecek et al., 2011) and then used the loci detected as variants for targeted genotype calling with the remaining individuals. First, a read pileup was performed for the 327 samples using the “bcftools mpileup” tool set to ignore indels, with a maximum depth of 1,000, and recalculated individual base alignment qualities (“redo‐BAQ”). Once the full pileup was produced, actual variants were called “bcftools call” set to the multiallelic‐caller mode and keeping all possible alternative alleles. All detected variant sites (*n* = 411,630) were used in a second round of “bcftools mpileup” for all individuals.

Filtering of the resulting vcf files was done in two rounds using VCFTOOLS v0.1.15 (Danecek et al., 2011). For the first round, all individuals were included and a minimum read depth of 6× and genotype quality score of 18 were required. Loci were filtered out if they were not present in at least 50% of individuals, had more than two alleles, had a mean depth of coverage greater than two standard deviations above the mean depth, or had a minor allele count less than 3. Loci were thinned to retain one site per 10,000 bp. Following this, the amount of missing data was assessed for each individual, and those with >60% missing were removed. Using the retained individuals, SNP filtering was repeated but starting again with all variants included. This time, loci were filtered out if they were not present in at least 70% of individuals, while maintaining the thresholds of remaining filters. Following this, we again assessed individual missing data and removed individuals with >50% missing data. Genotyping error rates were calculated by evaluating the number of mismatched genotypes between technical replicates (*n* = 16).

### 2.4 Population genetic analyses

Standard measures of genetic diversity, including observed and expected heterozygosity and G_IS,_ were calculated for each subpopulation using GENODIVE V 2.0b27 (Meirmans & Van Tienderen, 2004). Weir and Cockerham’s (1984) fixation index was calculated between all subpopulation pairs using the *hierfstat* package (Goudet, 2005) in the R statistical package, version 3.6.0 (R Development Core Team, 2010).

Evidence for population substructure was assessed using multiple methods. We used Bayesian clustering analysis, as implemented in STRUCTURE 2.3.4 (Pritchard, Stephens, & Donnelly, 2000). We evaluated from 1 to 10 clusters (*K*), with ten iterations of each, using a run length of 300,000 Markov chain Monte Carlo replicates after a burn‐in period of 100,000 and correlated allele frequencies under an admixture model with alpha set to 0.5. The most likely number of clusters was determined by plotting the log probability of the data (ln Pr(*X|K*)) across the range of *K* values tested and selecting the *K* value where the value of ln Pr(*X|K*) plateaued, as suggested in the STRUCTURE manual, and by calculating the deltaK statistic (Evanno, Regnaut, & Goudet, 2005) in STRUCTURE HARVESTER (Earl & vonHoldt, 2011). The 10 iterations were averaged using CLUMPP (Jakobsson & Rosenberg, 2007) to produce a single q‐matrix. A clustering analysis based on maximum likelihood implemented in ADMIXTURE (Alexander, Novembre, & Lange, 2009) was also used, with the optimal value of *K* determined using a cross‐validation procedure. We used default values and 10‐fold cross‐validation. We also used the model‐free discriminant analysis of principal components (DAPC; Jombart, Devillard, & Balloux, 2010) implemented in *Adegenet* (Jombart, 2008) in R. The best‐fit value of *K* was selected using the *find.clusters* function and Bayesian information criterion (BIC). The chosen value of *K* was based on the minimum number of clusters after which the BIC decreased by a negligible amount.

We used analysis of molecular variance (AMOVA, implemented in GENODIVE) to evaluate the proportions of total genetic variation that were contained within and among subpopulations, and within and among the genetic clusters suggested by population structure analyses.

To evaluate whether there are geographic regions where the strength of isolation‐by‐distance is higher‐than‐average or lower‐than‐average, we used EEMS (Petkova, Novembre, & Stephens, 2015), a method of visualizing variation in effective migration rates inferred from genetic dissimilarities. EEMS is based on a stepping‐stone model, where migration occurs between neighboring demes modeled in a dense regular grid (where the number of demes equals the number of spaces on the grid). The expected genetic dissimilarity between two individuals is calculated over all possible migration histories and routes between their deme on the grid, and migration rates for edges in the grid are adjusted so that the genetic differences expected under the model best match the observed differences in the data. These migration rates are interpolated across the grid to produce the “estimated effective migration surface.” In geographic areas with no samples, estimates cannot be calculated, and thus, in these regions, they are driven by the prior: no heterogeneity in migration rates (Petkova et al., 2015). In our dataset, this is particularly relevant in the geographic areas on the edges of our sampling distribution and in undersampled areas. Inputs required for this analysis are an outline of the area to be modeled, the geographic locations of samples, and a matrix of observed differentiation values. We generated this matrix based on the average pairwise differences between individuals using *bed2diffs_v2*, which imputes missing data based on the average genotype for that locus. We ran EEMS initially using the default values for proposal variances and other parameters and changed the values in subsequent runs until the frequency of accepting proposals of each type was between 10% and 40%, as suggested in the software documentation. Using the optimized set of parameters, we ran five independent chains for 10,000,000 MCMC iterations, with a burn‐in of 2,000,000, and thinning every 9,999 iterations. We assessed convergence of these chains and plotted the combined EEMS results using the R package *rEEMSplots*. The resulting contour plot was overlaid with a map of Canada using the R packages *rworldmap* and *rworldxtra* (South, 2011).

### 2.5 Assignment accuracy

We estimated assignment accuracies across polar bear subpopulations using principal component analyses and Monte Carlo cross‐validation procedures implemented in the *AssignPOP* package (Chen et al., 2018) in R. We investigated the assignment accuracy results of a predictive model built using a support vector machine (model = svm) classification, based on training sets using the most informative 75% of loci and a randomly sampled 75% of individuals. The rate of assignment was tested using the 25% of individuals that has been left out of training, and then averaged across 30 iterations. Assignment tests were only performed for the nine subpopulations with a sample size greater than 10.

## 3 RESULTS

The final dataset consisted of 358 individuals (Table S1), that met genotype depth and missing data filters, plus 16 technical replicates, genotyped at 13,488 loci. The average depth was 27×, with an average of 13% missing data within individuals. The number of discordant genotypes between technical replicates ranged from 11 to 31, with an overall genotyping error rate estimated to be 0.2%.

The subpopulations all had very similar levels of genetic diversity (Table 1). For example, expected heterozygosity ranged from 0.17 to 0.19, while *G*_IS_ values were all slightly negative and ranged from −0.08 to −0.01. *F*_ST_ values were all less than 0.1 (Tables S2 and S3).

**FIGURE 2:**
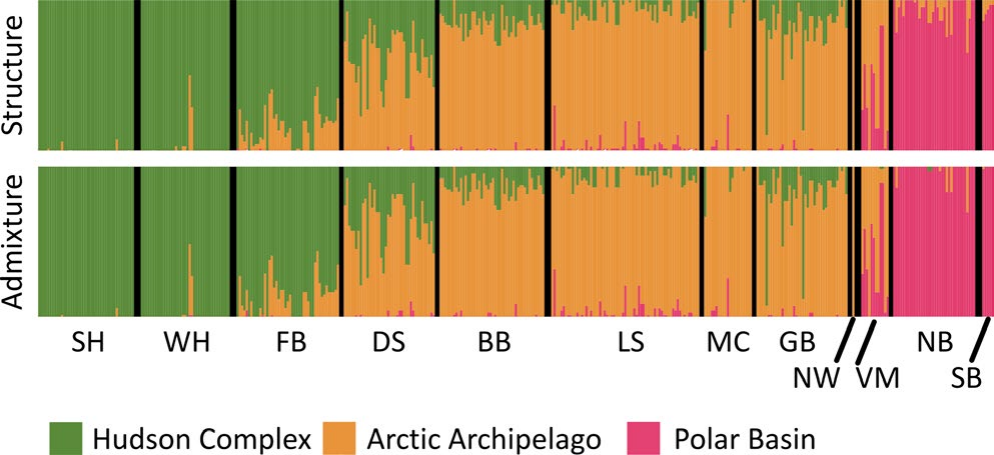
Genetic cluster assignment bar plots for *K* = 3 from STRUCTURE and ADMIXTURE. The three genetic clusters are identified with different colors. Each individual is represented as a bar, with the proportion of the bar each color representing their assignment to the genetic clusters. Subpopulation abbreviations are as in Table 1

Similar patterns were resolved across the three population cluster analysis methods. The ln Pr(*X|K*) for STRUCTURE plateaued around *K* = 3 (Figure S1a), with the highest level of deltaK at *K* = 2 (Figure S1b), the lowest cross‐validation error scores in ADMIXTURE were for *K* = 3 (Table S3), and for the find.clusters DAPC analysis, the lowest BIC score was *K* = 2 (Figure S2), with *K* = 3 only having a slightly higher value. Over all analyses, the groupings at *K* = 3 correspond to geographic areas that we hereafter refer to as the “Hudson Complex,” “Arctic Archipelago,” and “Polar Basin” (Figure 2). Barplots showing *K* = 2, 4, and 5 can be found in Figure S3.

Most individuals show a high assignment to one genetic cluster. Only 34 individuals in our sample of 358 have a membership *Q*‐value of less than 0.7 to a cluster (depicted as black dots on the map in Figure 1). In the clustering analyses, the individuals within a subpopulation tend to have majority assignment to the same cluster, with the exception of Davis Strait (DS), which has individuals with high assignment to both the Hudson Complex and Arctic Archipelago, and Viscount Melville (VM), which has individuals with high assignment probabilities to the Arctic Archipelago and Polar Basin. However, for the AMOVA, we included DS and VM in the Arctic Archipelago cluster because most individuals were assigned to that group. Most variation was among individuals (0.994), with much smaller, but significant levels of variation partitioned among subpopulations within the clusters (0.007) and among clusters (0.022).

**FIGURE 3:**
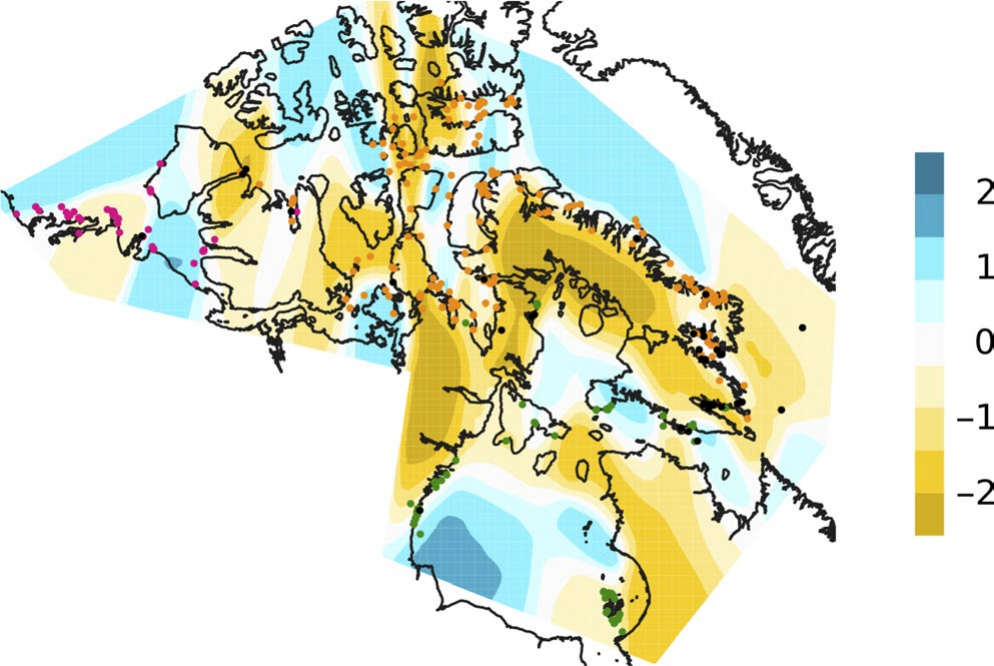
The EEMS contour plot of effective migration rates. The scale is log10(migration), relative to the overall migration rate across the modeled area. Points are the sampling locations of the polar bears, colored following Figure 1. Areas at the dark yellow end of the spectrum exhibit on average higher‐than‐average isolation‐by‐distance

The EEMS analysis clearly showed regions where isolation‐by‐distance is stronger than the average rate (Figure 3). Results are consistent with previous analyses, identifying less migration than expected based on the null expectation of isolation‐by‐distance (IBD) in areas separating the three genetic clusters. Many of the regions identified as having less migration (i.e., higher‐than‐average IBD) appear to correspond with areas with more contiguous terrestrial habitats, while at least some areas of high migration lie along shoreline or marine areas with islands (e.g., King William Island, or between NW Baffin Island and Somerset Island), although some areas identified as having high migration lie in obvious sampling gaps.

The results from the assignment tests (Table 1) showed that some subpopulations are genetically distinguishable, with self‐assignment rates the highest in the Northern Beaufort Sea (NB, 0.93).

## 4 DISCUSSION

In this study, we used thousands of genome‐wide SNP markers to assess population structure across the range of polar bears in Canada. Our results clearly show differentiation within Canada, and the membership patterns and geographic ranges of the “Hudson Complex,” “Arctic Archipelago,” and “Polar Basin” genetic groups match closely with the results of previous studies using microsatellite loci (Malenfant et al., 2016; Paetkau et al., 1999; Peacock et al., 2015) and a preliminary analysis using ~3,000 SNPs and 78 individuals (Malenfant et al., 2015). The previous studies included the global range of polar bears, and found that the genetic cluster of the Beaufort Sea continues around the arctic coast of Asia and Europe to the eastern side of Greenland (Malenfant et al., 2016; Paetkau et al., 1999; Peacock et al., 2015), which is why we have chosen to refer to this cluster in our study as the Polar Basin. A fourth genetic cluster has been identified by previous authors (Malenfant et al., 2015, 2016; Paetkau et al., 1999) coincident with the Norwegian Bay subpopulation, but due to our very small sample size (*n* = 1), we cannot confirm its existence.

Four ecoregions are recognized within the global range of polar bears, based on sea ice dynamics (i.e., seasonality of sea ice) and the associated differences in polar bear life history (Amstrup, Marcot, & Douglas, 2013). The genetic clusters do not entirely align with these ecoregions; for example, some of the subpopulations in the Arctic Archipelago genetic cluster are in the Seasonal Ice ecoregion, while others are in the Archipelago ecoregion. Thus, the ecoregions (based on sea ice dynamics) are not the sole drivers of population structuring, and other finer‐scale processes may be at play.

In the EEMS analysis, areas with reduced migration generally corresponded to landmasses, such as Baffin Island (Figure 3), which may highlight land as being a natural barrier to polar bear movement. The narrow Fury and Hecla Strait between Baffin Island and the Melville Peninsula also seems to restrict gene flow between the Arctic Archipelago and Hudson Complex genetic clusters, which is highlighted in the EEMS analysis (Figure 3) as being a region where effective migration is substantially lower than the average isolation‐by‐distance. The eastern boundary between the Arctic Archipelago and Hudson Complex is not clearly defined, with a number of admixed individuals sampled around the southeastern end of Baffin Island. Within the Hudson Bay, there is suggested to be higher‐than‐average estimated effective migration, which may reflect the seasonal mixing of bears on the sea ice in the bay or be a consequence of the distribution of our samples on opposite coastlines. Differentiation between the Polar Basin and Arctic Archipelago seems to be the result of gene flow being restricted along the straits south and north of Victoria Island. Within the Arctic Archipelago, there are regions where migration appears reduced. The genetic structure evident in such a mobile species that uses sea ice for foraging and movement may reflect variation in marine productivity (e.g., fidelity to coastal areas overlying continental shelf where prey are abundant) and in sea ice quality and seasonality (Hamilton & Derocher, 2019).

There are no hard boundaries between the genetic clusters, as evidenced by individuals with mixed membership to clusters, and individuals with high membership to a cluster being sampled outside the general geographic boundaries of that cluster. Monitoring the frequency of admixture and dispersal among the genetic clusters and the temporal stability of their distributions will help to understand the impacts of changing environmental conditions on polar bear behavior in the future. Polar bears have extremely high dispersal capabilities demonstrated by their vast home range which can span up to ~400,000 km^2^ (Auger‐Méthé, Lewis, & Derocher, 2016) and long‐distance swimming capabilities (Pagano et al., 2012). Future research should augment the geographic intensity of sampling and use landscape genetic approaches and climate models (e.g., Mioduszewski, Vavrus, Wang, Holland, & Landrum, 2019) to better understand possible outcomes of changing climates on polar bears.

The results of our study, along with previous findings, suggest that subpopulations are not distinct evolutionary entities (e.g., Viengkone et al., 2016; Vongraven, Derocher, & Bohart, 2018). There is low *F*_ST_ among subpopulations, as well as very little variation partitioned among them in the AMOVA. However, there is clearly some underlying genetic structure that allows individuals to be assigned to the subpopulation where they were sampled with relatively high accuracy in the *AssignPOP* analysis, for example, Lancaster Sound (LS) and Baffin Bay (BB). Although imperfect, our ability to correctly assign individual bears to the subpopulation of origin may have management implications (e.g., assessing identity of individuals from different subpopulations that are mixing in foraging areas on ice). Levels of genetic diversity, as measured by observed and expected heterozygosity, do not vary markedly across the subpopulations (Table 1), which is consistent with previous studies (Malenfant et al., 2016; Paetkau et al., 1999; Peacock et al., 2015). This homogeneity in estimates of genetic diversity is despite the fact that subpopulations differ markedly in estimated census population size (161 to 2,826—references within Hamilton & Derocher, 2019) and density (e.g., 0.57 to 9.30 individuals per km^2^—Hamilton & Derocher, 2019), and different regions having experienced divergent environmental and human hunting histories (COSEWIC, 2008).

Our initial goal was to mitigate ascertainment bias by genotyping a balanced sample of individuals from each subpopulation, but ultimately this was not possible due to issues with poor DNA quality, and limited sampling in subpopulations where less harvesting occurs. Harvesting is intentionally skewed toward male bears, and our sample reflects that, with 62% of samples on average within a subpopulation and 65% of samples overall being male. Male‐skewed sex ratio in our sample should not impact conclusions regarding population structure. Taylor et al. (2001) found no differences in distances moved between sexes in six more northern Arctic polar bear subpopulations. Further, Campagna et al. (2013) found no evidence of sex‐biased gene flow based on analysis of microsatellites. We were conscious of the need to include samples that were collected in as close to the same period of time as possible. Previous range‐wide studies have largely used samples collected during population surveys, and thus, sampling was highly asynchronous across subpopulations. To determine whether this impacted their results, Peacock et al. (2015) tested whether they could detect genetic differentiation among sampling periods separated by decades within subpopulations. While they found no differences, this result could be due to lack of sensitivity in their markers, or associated with other problems with their dataset highlighted by Malenfant et al. (2016).

To improve our understanding of how polar bears are responding to climate change, there is interest in developing new monitoring tools that complement existing methods. In Canada, polar bear monitoring has been evolving in response to new technologies and concerns about the invasiveness of monitoring activities. In recent years, physical capture of individuals for capture–mark–recapture (CMR) studies has been minimized in favor of aerial genetic biopsy CMR to estimate abundance (Pagano, Peacock, & McKinney, 2014, Scientific Working Group to the Canada‐Greenland Joint Commission on Polar Bear, 2016). However, these surveys are costly, and only relatively small portions of the entire polar bear range are being surveyed at any time, with many years or decades between successive surveys for some subpopulations (Durner et al., 2018; Hamilton & Derocher, 2019; Vongraven & Peacock, 2012). This limitation means that such surveys alone cannot provide contemporaneous assessment of populations across the range, and may not be sensitive enough to detect the rapid changes in polar bear population structure that are expected to accompany environmental changes (Hamilton & Derocher, 2019; Laidre et al., 2015; Vongraven & Peacock, 2012). One solution may be to use noninvasively collected scat for genetic CMR and observation of population structure.

Previous attempts at using scat for polar bear monitoring have used DNA microsatellite markers, with a relatively low genotyping success rate of 43% (P.V.C.D.G., unpublished data). New methods using single nucleotide polymorphism (SNP) markers may allow a higher rate of successful genotyping (Campbell, Harmon, & Narum, 2015; Kleinman‐Ruiz et al., 2017). The shorter length of SNP‐containing DNA fragments (~50–70 bp relative to microsatellites (~80–300 bp)) could accommodate genotyping of low quantity and quality DNA and thus mitigate common challenges to genotyping noninvasive samples (Fitak, Naidu, Thompson, & Culver, 2016; Kleinman‐Ruiz et al., 2017; von Thaden et al., 2017). Other advantages of SNPs over microsatellites for genotyping of noninvasive samples include lower mutation rates, clearer mutation patterns, and greater standardization and automation potential (Morin, Luikart, Wayne, the SNP workshop group, 2004; Olsen et al., 2011). One current limitation to deploying such a SNP‐based monitoring scheme is the absence of high‐quality, geographically representative baseline data from which to select markers, and analyze and interpret future genetic results. From our dataset of SNPs presented here, a subpanel of highly informative loci could be selected for identifying recaptures and assigning parentage (Anderson & Garza, 2006; Andrews et al., 2018) and enable a noninvasive monitoring program using feces or hair snags.

## 5 CONCLUSIONS

Within the Canadian range of polar bears, subpopulation structure is present and has been consistently recovered across datasets and genetic markers. There is congruence between our results based on thousands of genome‐wide SNPs and previous studies using 16–21 microsatellite or ~3,000 SNP loci. Our samples have not been included in any previous studies (except for three which were also used by Peacock et al., 2015) and provide independent corroboration of major genetic patterns. Thus, between mutually exclusive datasets of individuals and genetic markers when compared to previous studies, we have reconstructed the same understanding of population differentiation and genetic diversity patterns. We do have some sampling gaps in southern extent of Hudson Bay, southern Davis Strait, and northern Quebec, with the former two populations reported to exhibit modest genetic differentiation (Crompton, Obbard, Petersen, & Wilson, 2008; Malenfant et al., 2016; Peacock et al., 2015). Regardless, our now firm baseline understanding of population structure will be critical to our ability to accurately assess the effects of climate change and sea ice loss on connectivity, and measure the subsequent impacts on demography and evolutionary dynamics in polar bears. From these data, a subpanel of SNP markers can be selected for use in future studies to monitor changes in population structure from both high‐ and low‐quality DNA sources, including tissue samples from aerial biopsy sampling and the annual harvest, or noninvasively collected hair from snags, or scat collected on the land.

## CONFLICT OF INTEREST

None declared.

## AUTHOR CONTRIBUTIONS

E.L.J., P.V.C.D.G., and S.L.C. conceived and designed the study; M.B. and M.D. facilitated access to the tissue samples and provided insight on polar bear ecology and management; E.L.J., C.T., R.C.C., and K.M.H. collected genomic data and performed analyses; and E.L.J., C.T., and S.C.L. wrote the first version of the manuscript with all authors contributing to revisions.

## Supporting information

Supplementary Material

Table S1

## Data Availability

The ddRAD sequence data are available as individual BAM files through the NCBI Sequence Read Archive as BioProject PRJNA602523. The filtered vcf file used for analyses is available on Dryad at https://doi.org/10.5061/dryad.gb5mkkwkw.
