# Supplementary material for: Canadian polar bear population structure using genome‐wide markers
Source: Ecol Evol. 2020 Mar 24;10(8):3706–14. doi: 10.1002/ece3.6159 (PMC7160183; doi:10.1002/ece3.6159)
Supplement: Supplementary file 1 — Supplementary Material [file ECE3-10-3706-s001.docx]

**Supplemental Material for: Canadian polar bear population structure using genome-wide markers**

Evelyn L. Jensen, Christina Tschritter, Peter V. C. de Groot, Kristen M. Hayward, Marsha Branigan, Markus Dyck, Rute Clemente-Carvalho and Stephen C. Lougheed


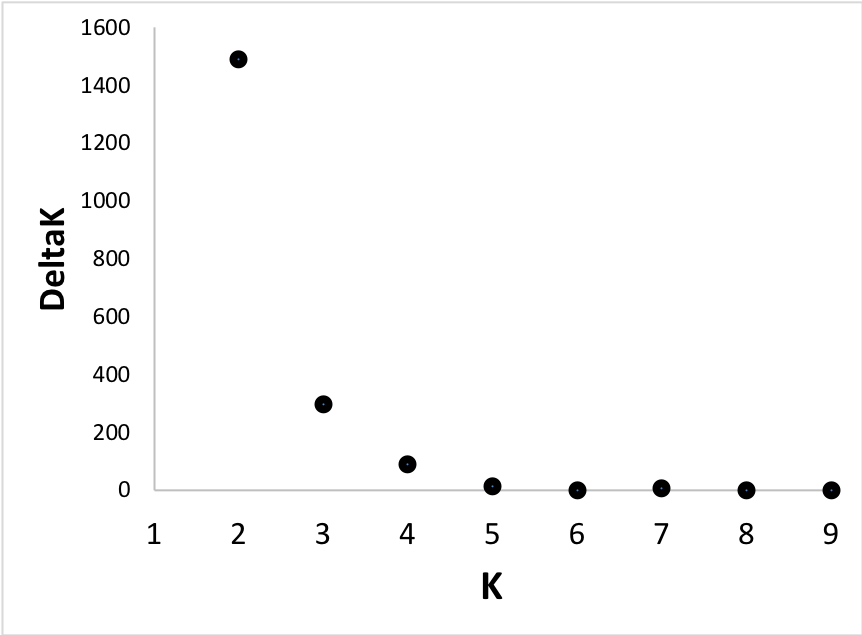


**A**

**B**

Supplemental Figure 1. Plot of A) ln P(*K*) and B) deltaK for each number of clusters (K) evaluated in the STRUCTURE analysis.


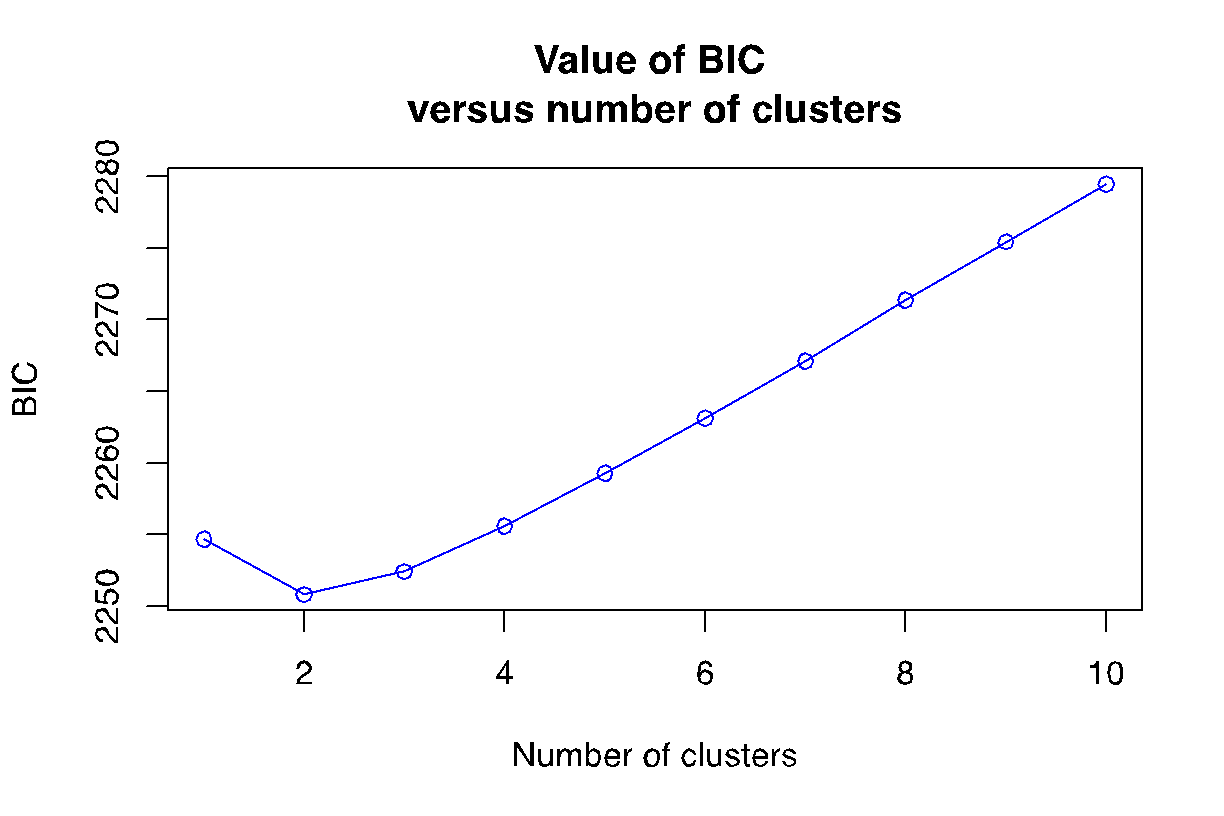


Supplemental Figure 2. Plot of Bayesian Information Criterion (BIC) from the Discriminant Analysis of Principal Components (DAPC) analysis depicting the support for each number of clusters evaluated.

Supplemental Figure 3. Genetic cluster assignment bar plots for K=2, K=4 and K=5 from STRUCTURE and ADMIXTURE. The genetic clusters are identified with different colors. Each individual is represented as a bar, with the proportion of the bar each color representing their assignment to the genetic clusters. Subpopulation abbreviations are as in Table 1.

*For supplemental Table 1, see attached Excel workbook.

Supplemental Table 2. Values of pairwise Weir and Cockerham (1984) F_ST_ between subpopulations based on the dataset of SNPs. F_ST_ was not calculated for NW due to its sample size of 1. Abbreviations follow Table 1. Cells shaded darker colors are of greater magnitude.

|  | BB | DS | FB | GB | LS | MC | NB | SB | SH | VM |
| --- | --- | --- | --- | --- | --- | --- | --- | --- | --- | --- |
| DS | 0.01 |  |  |  |  |  |  |  |  |  |
| FB | 0.02 | 0.01 |  |  |  |  |  |  |  |  |
| GB | 0.01 | 0.01 | 0.02 |  |  |  |  |  |  |  |
| LS | 0.01 | 0.01 | 0.03 | 0.01 |  |  |  |  |  |  |
| MC | 0.02 | 0.02 | 0.04 | 0.01 | 0.01 |  |  |  |  |  |
| NB | 0.03 | 0.04 | 0.06 | 0.03 | 0.03 | 0.04 |  |  |  |  |
| SB | 0.03 | 0.03 | 0.05 | 0.03 | 0.02 | 0.04 | 0 |  |  |  |
| SH | 0.04 | 0.03 | 0.01 | 0.04 | 0.04 | 0.06 | 0.07 | 0.07 |  |  |
| VM | 0.02 | 0.03 | 0.05 | 0.02 | 0.01 | 0.01 | 0.02 | 0.02 | 0.06 |  |
| WH | 0.03 | 0.02 | 0.01 | 0.03 | 0.04 | 0.05 | 0.07 | 0.06 | 0 | 0.06 |

Supplemental Table 3. Cross-validation error scores from ADMIXTURE analysis of the probability of a given number of clusters (K).

| **K** | **Error Score** |
| --- | --- |
| **1** | 0.34895 |
| **2** | 0.33802 |
| **3** | 0.33610 |
| **4** | 0.33625 |
| **5** | 0.33828 |
| **6** | 0.34127 |
| **7** | 0.34412 |
| **8** | 0.34930 |
| **9** | 0.35136 |
| **10** | 0.35772 |
